# Multiplex real-time PCR assay to detect illegal trade of CITES-listed shark species

**DOI:** 10.1038/s41598-018-34663-6

**Published:** 2018-11-05

**Authors:** Diego Cardeñosa, Jessica Quinlan, Kwok Ho Shea, Demian D. Chapman

**Affiliations:** 1 School of Marine and Atmospheric Science, Stony Brook University, Stony Brook, New York, 11794 United States of America; 2 Fundación Colombia Azul, Bogotá, Colombia; 3 Department of Biological Sciences, Florida International University, 3000 NE 151st Street, North Miami, Florida, 33181 United States of America; 4 BLOOM Association, Central, Hong Kong

## Abstract

The Convention on International Trade in Endangered Species of Wild Fauna and Flora (CITES) is a multilateral environmental agreement to ensure that the international trade of threatened species is either prohibited (Appendix I listed species) or being conducted legally, sustainably, and transparently (Appendix II listed species). Twelve threatened shark species exploited for their fins, meat, and other products have been listed under CITES Appendix II. Sharks are often traded in high volumes, some of their products are visually indistinguishable, and most importing/exporting nations have limited capacity to detect illicit trade and enforce the regulations. High volume shipments often must be screened after only a short period of detainment (e.g., a maximum of 24 hours), which together with costs and capacity issues have limited the use of DNA approaches to identify illicit trade. Here, we present a reliable, field-based, fast (<4 hours), and cost effective ($0.94 USD per sample) multiplex real-time PCR protocol capable of detecting nine of the twelve sharks listed under CITES in a single reaction. This approach facilitates detection of illicit trade, with positive results providing probable cause to detain shipments for more robust forensic analysis. We also provide evidence of its application in real law enforcement scenarios in Hong Kong. Adoption of this approach can help parties meet their CITES requirements, avoiding potential international trade sanctions in the future.

## Introduction

The Convention on International Trade in Endangered Species of Wild Fauna and Flora (CITES) is a binding and multilateral environmental agreement with the objective of prohibiting trade of wildlife species in immediate danger of extinction (Appendix I listing) and preventing additional threatened species reaching this critical stage by regulating trade so that it is traceable, legal, and sustainable (Appendix II listing). CITES is the first line of defense against illegal wildlife trafficking, with 183 parties (i.e. signatory nations), and lists over 35,000 animal and plant species^1^. The vast majority of these species (96%) are listed in Appendix II and exporting parties have the obligation to document that traded specimens are traceable through the supply chain, were legally obtained, and that trade it is not detrimental for the survival of the species^2^. Importing parties are required to monitor imports and certify that incoming specimens are accompanied by the appropriate documentation^2^. Failure to properly implement CITES for any listed species can eventually lead to international trade sanctions being placed on offending parties^2^.

Sharks are a group of marine fish many of which are threatened by overexploitation to satisfy demand for internationally traded products, primarily fins (for use in luxury Asian soup dishes) and meat^3,4^. Twelve shark species have been listed in CITES Appendix II to date. The first round of shark listings took place from 2001–2004, with the whale shark *Rhincodon typus* (2001), basking shark *Cetorhinus maximus* (2001), and great white shark *Carcharodon carcharias* (2004). These species all share the characteristics of being iconic, extremely large bodied (>4.0 m total length at maturity), and some products from these species (e.g., fins, dressed carcasses) can be readily identified by their very large sizes (except when procured from juvenile stages) or other morphological features (e.g., jaws and teeth for great whites). These species also share the characteristic that Appendix II listing is not as stringent as the domestic protection these species receive in many jurisdictions (i.e., landing and trade prohibition^4,5^). Products of these species have generally not been detected in post-listing studies of major shark fin and meat markets, suggesting they are rare in trade^6–10^.

The second round of shark listings took place from 2013–2016, where the porbeagle shark *Lamna nasus* (2013), scalloped hammerhead *Sphyrna lewini* (2013), great hammerhead *S*. *mokarran* (2013), smooth hammerhead *S*. *zygaena* (2013), the oceanic whitetip shark *Carcharhinus longimanus* (2013), silky shark *C*. *falciformis* (2016), bigeye thresher shark *Alopias superciliosus* (2016), pelagic thresher shark *A*. *pelagicus* (2016), and common thresher shark *A*. *vulpinus* (2016) were added to Appendix II. This group of species all share the characteristics of being smaller at maturity than white, whale, or basking sharks, and being much more common in trade both pre and post-CITES listing^9,10^. The silky, scalloped hammerhead, and smooth hammerhead represent the second, fourth, and fifth most common species in the contemporary fin trade^9,10^. Some of the dried unprocessed fins (e.g., first dorsal, pectoral) and dressed carcasses of these sharks can be identified using morphological characters^11,12^. Trade in meat, other processed products, and some, less distinctive fin types (e.g., lower caudal) are more challenging to detect without the aid of genetic testing. The Secretariat of CITES recently recommended that parties “share experiences with, and knowledge of, forensic means to efficiently, reliably and cost- effectively identify shark products in trade” (SC69 Doc. 50), including genetic methods.

It is evident that enforcing CITES regulations is a major challenge, in part due to limited availability of rapid, reliable, and cost-effective tools to identify traded products^3^, especially for the species listed from 2013–2016^10^. Wildlife forensics tools, such as DNA barcoding^13–15^ and PCR-based species-specific assays^16–18^, are the most reliable and widely applicable species identification approaches available to assess and detect illicit activities associated with wildlife trade^19–21^. However, the application of these tools to detect CITES listed species is difficult when inspections must be conducted in a short time frame (e.g. less than 24 hours as is the legal requirement in some locations, such as the United States of America, Hong Kong Special Administrative Region of China), with limited scope for randomly sampling unknown products, transferring tissue samples to a laboratory, and going through all of the steps required for DNA barcoding (i.e., DNA isolation, PCR, product purification, sequencing^19^) or using available species-specific PCR tests (i.e., DNA isolation, testing for CITES listed species contained in up to four separate multiplex PCR assays, gel electrophoresis^19^). This is especially problematic given that individual shipments of shark products can be quite large (i.e., 10^2^–10^3^ kg^20^). Real-time PCR (rtPCR) techniques are an alternative tool that reduce the number of steps to species diagnosis and offers the advantage over DNA barcoding of being able to be conducted outside of the laboratory. Typically, rtPCR techniques have been developed for the quantification of nucleic acids^21^, and for the detection of pathogen microorganisms^22–24^. This technique uses target-specific primers and fluorescent dyes (e.g. SYBR Green, TaqMan probes) to detect if PCR amplification occurred due to the presence of the targeted nucleic acid template, eliminating the need for sequencing and agarose gels, and enabling a reliable identification of targeted species in real time. In addition, the last stage of rtPCR protocols usually generates melt curves and melt curve temperatures, that can further be used for the identification of the target species/locus^25,26^. Melt curves are generated by the dissociation of double-stranded DNA during heating, and melt curve temperatures typically vary with the GC/AT ratio of the generated amplicon and its size^26^.

Here, we present a rtPCR protocol capable of rapidly detecting the presence of nine of the twelve CITES-listed shark species in a single multiplex PCR that could be used as a field-based tool to detect the illegal exportation/importation of these species (i.e. cases of import/export without the correct documentation). The multiplex includes all of the commercially important sharks listed from 2013–2016, with the exception of the oceanic whitetip shark. While the test itself does not discriminate which of the species is present (with a few exceptions based on melt curve analysis) it rapidly and cost effectively (~4 hours for 95 samples; $0.94 USD per sample) provides sufficient information to justify holding shipments based on the illegal presence of CITES listed species. This would provide time for more rigorous forensic testing to verify species-of-origin to meet typical evidentiary standards required for prosecution. We also present three field tests conducted in Hong Kong to inspect the contents of a shark fin consignment in collaboration with local authorities that provide proof-of-concept of the portability of this approach.

## Methods

**Figure 1:**
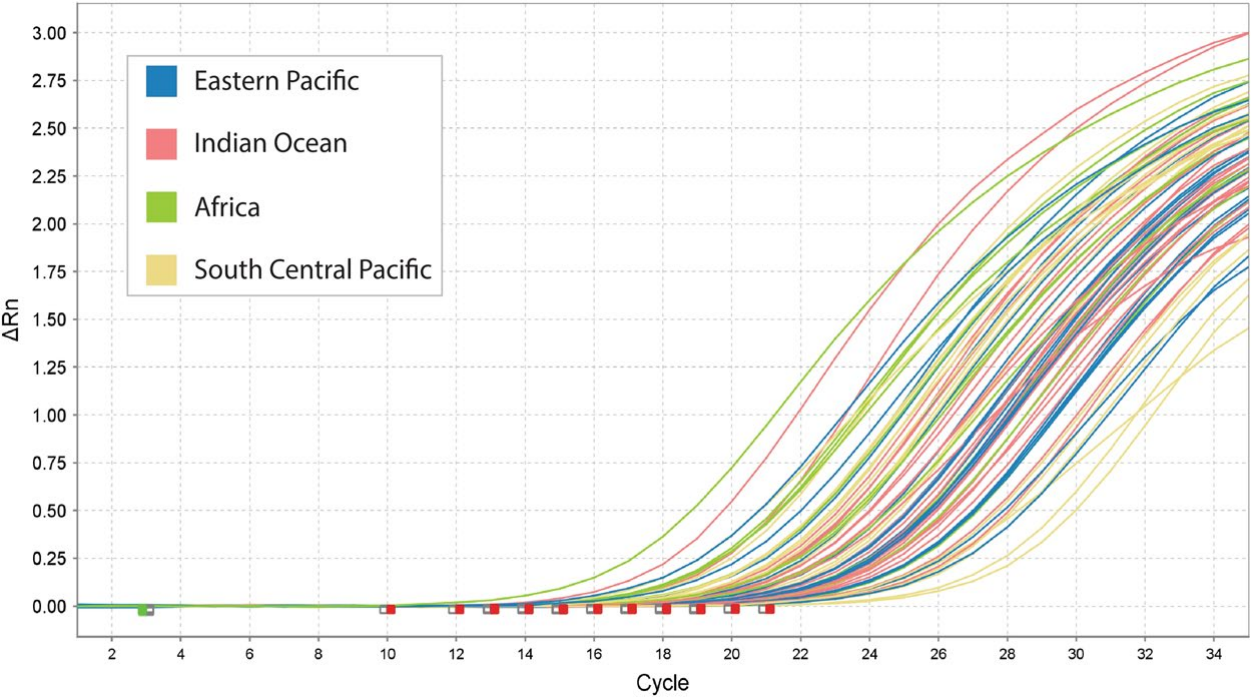
Amplification plot of silky shark samples from around the world using the new species-specific primer for the silky shark.

Previously published species-specific primers for CITES-listed species^16,17,27,28^ were used as a starting point for our multiplex rtPCR assay. The original published studies validated these primers against large, geographically broad samples of the target species, and demonstrated that the primer failed to amplify a large number of non-target species including all or most of the target species’ close relatives (i.e. congeners) through agarose gel electrophoresis^18,29^.Originally, published primers for hammerhead and thresher shark species were tested on 56 species^17,28^, great white shark primers on 68 species^16^, and porbeagle and silky shark primers on 34 species^27^. Initially, all published primers for these CITES listed species (Table 1) were tested individually with one universal reverse primer against 48 target and non-target species comprising six orders and twelve families (Table 2) to ensure complete specificity of each individual primer when adapted to SYBR Green chemistry. Each 20 μL reaction included, 10 μL of PowerUp SYBR Green Master Mix (Applied Biosystems), 5 μL of primer mix (equal mixture of primers from 10 μM stock solutions), and 5 μL of extracted DNA. DNA extractions were either conducted from dried unprocessed fin, meat, or wet unprocessed fins using Chelex DNA extraction protocols^30,31^, where a small sample (∼2 mm^2^) was added to a PCR tube containing 200 μL of 10% Chelex solution and heated for 20 minutes at 60 °C and for 25 minutes at 99 °C, followed by a brief centrifugation and storage at 4 °C. A no-template negative control was included in every extraction and rtPCR batch to control for reagent contamination. Species-specific amplifications were tested against a wide range of annealing temperatures (60°–70 °C) on the QuantStudio5 System (Thermofisher) to determine the most stringent temperature that could be used to amplify the target species and an annealing temperature of 69 °C was selected for further trials. Early trials showed that the silky shark primer, described by^27^, did not amplify well in a rtPCR format. It also frequently amplified genomic DNA from the spinner shark (*C*. *brevipinna*). Thus, we designed a novel species-specific primer for silky sharks (Table 1). We aligned all reference sequences for the internal transcribed spacer 2 (ITS2) deposited in GeneBank by^27^ in SeaView v4^32^, and designed a putative species-specific primer based on nucleotide differences in this locus. The ITS2 locus was chosen as the target locus for silky sharks because (i) it is highly conserved within shark species, (ii) contains enough nucleotide polymorphisms to achieve species-specific diagnosis and sequence differences between distantly related species are sufficiently large to preclude meaningful alignments, and (iii) all other species-specific primers in our multiplex were designed for this particular locus, thus allowing us to use the same universal reverse primer to amplify the novel primer^16,17,27,28^. This novel silky shark primer was individually tested for amplification performance in the rtPCR format with silky shark tissues collected over a wide geographic area (Fig. 1) and against all test samples in Table 2. Once we had validated this silky primer, all species-specific primers and one universal reverse primer were combined into a single multiplex of 20 μL including 10 μL of PowerUp SYBR Green Master Mix (Applied Biosystems), 5 μL of primer mix (equal mixture of all primers from 10 μM stock solutions), and 5 μL of extracted DNA. This final multiplex was tested against all test species (Table 2) to detect possible primer interaction that could obstruct the amplification. The thermal cycle profile is shown in Supplementary Fig. S1. Our expected result was that genomic DNA from the nine CITES listed species would amplify and enable SYBR green to fluoresce, while DNA from any other species would not.

**Table 1:** Primers used for the multiplex rtPCR assay to detect CITES-listed species.

Primer name	N tested rtPCR	N tested original study	Amplicon size (bp)	Primer sequence (5′-3′)
FISH 28 S R^d^				TCC TCC GCT TAG TAA TAT GCT TAA ATT CAG C
ScHH401 F^a^	15	140	445	GGT AAA GGA TCC GCT TTG CTG GA
SmHH630 F^a^	15	41	249	TGA GTG CTG TGA GGG CAC GTG GCC T
GtHH123 F^a^	15	45	782	AGC AAA GAG CGT GGC TGG GGT TTC GA
rtSilky F^b^	96	n/a	328	TCT CTC CCC CCC CCT CAC TCT GCC
GWSITS2 F^c^	15	52	580	GCT GGA GTT CAT TCT CCG TGC TG
Porbeagle F^d^	5	18	554	GTC GTC GGC GCC AGC CTT CTA AC
Bigeye Thresher 272 F^e^	15	20	1000	AGT GCT TGA CCA TTC GGT GTG CGT
Common Thresher 1048 F^e^	15	32	385	CCG GCC ATG CCT TCT GCA ACT TG
Pelagic Thresher 1113 F^e^	15	18	230	CAA GCC TTG CAC TTT CGA ATG AAG C

^a^Abercrombie *et al*.^17^.

^b^This study, ^c^Chapman *et al*.^16^ .

^d^Shivji *et al*.^27^ .

^e^Abercrombie *et al*.^28^.

**Table 2:** Shark samples (unprocessed and processed) used to validate the multiplex rtPCR assay.

Order	Family	Species	# of samples (unprocessed/processed)
Carcharhiniformes	Sphyrnidae	Sphyrna lewini*	15/10
Carcharhiniformes	Sphyrnidae	Sphyrna zygaena*	15/10
Carcharhiniformes	Sphyrnidae	Sphyrna mokarran*	15/10
Carcharhiniformes	Carcharhinidae	Carcharhinus falciformis*	96/10
Lamniformes	Alopiidae	Alopias pelagicus*	15/10
Lamniformes	Alopiidae	Alopias superciliosus*	15/7
Lamniformes	Alopiidae	Alopias vulpinus*	15/4
Lamniformes	Lamnidae	Lamna nasus*	5/9
Lamniformes	Lamnidae	Carcharodon carcharias*	15/0
Carcharhiniformes	Carcharhinidae	Carcharhinus longimanus**	15/0
Carcharhiniformes	Sphyrnidae	*Sphyrna tiburo*	15/0
Carcharhiniformes	Carcharhinidae	*Galeocerdo cuvier*	15/0
Carcharhiniformes	Carcharhinidae	*Carcharhinus limbatus*	15/0
Carcharhiniformes	Carcharhinidae	*Carcharhinus amblyrhynchoides*	0/2
Carcharhiniformes	Carcharhinidae	*Carcharhinus plumbeus*	15/0
Carcharhiniformes	Carcharhinidae	*Carcharhinus signatus*	5/0
Carcharhiniformes	Carcharhinidae	*Carcharhinus obscurus*	2/0
Carcharhiniformes	Carcharhinidae	*Carcharhinus leucas*	15/0
Carcharhiniformes	Carcharhinidae	*Carcharhinus perezi*	15/0
Carcharhiniformes	Carcharhinidae	*Carcharhinus altimus*	15/0
Carcharhiniformes	Carcharhinidae	*Carcharhinus brachyurus*	10/0
Carcharhiniformes	Carcharhinidae	*Carcharhinus brevipinna*	15/0
Carcharhiniformes	Carcharhinidae	*Carcharhinus melanopterus*	6/0
Carcharhiniformes	Carcharhinidae	*Carcharhinus amblyrhynchos*	7/0
Carcharhiniformes	Carcharhinidae	*Carcharhinus dussumieri*	0/4
Carcharhiniformes	Carcharhinidae	*Carcharhinus sorrah*	8/0
Carcharhiniformes	Carcharhinidae	*Carcharhinus isodon*	2/0
Carcharhiniformes	Carcharhinidae	*Carcharhinus porosus*	4/0
Carcharhiniformes	Carcharhinidae	*Prionace glauca*	15/0
Carcharhiniformes	Carcharhinidae	*Carcharhinus acronotus*	5/0
Carcharhiniformes	Carcharhinidae	*Carcharhinus albimarginatus*	5/0
Carcharhiniformes	Carcharhinidae	*Carcharhinus galapagensis*	5/0
Carcharhiniformes	Carcharhinidae	*Carcharhinus amboinensis*	5/0
Carcharhiniformes	Carcharhinidae	*Carcharhinus porosus*	5/0
Carcharhiniformes	Carcharhinidae	*Rhizoprionodon terraenovae*	10/0
Carcharhiniformes	Carcharhinidae	*Negaprion brevirostris*	10/0
Carcharhiniformes	Triakidae	*Mustelus canis*	5/0
Carcharhiniformes	Triakidae	*Galeorhinus galeus*	5/0
Carcharhiniformes	Hemigaleidae	*Hemipristis elongata*	5/0
Orectolobiformes	Ginglymostomidae	*Ginglymostoma cirratum*	10/0
Squaliformes	Centrophoridae	*Centrophorus niaukang*	3/0
Squaliformes	Centrophoridae	*Centrophorus granulosus*	5/0
Squaliformes	Centrophoridae	*Centrophorus owstoni*	2/0
Squaliformes	Squalidae	*Squalus cubensis*	4/0
Lamniformes	Lamnidae	*Isurus oxyrinchus*	15/0
Lamniformes	Lamnidae	*Lamna ditropis*	2/0
Lamniformes	Odontaspidae	*Carcharias taurus*	10/0
Hexanchiformes	Hexanchidae	*Hexanchus griseus*	3/0
Hexanchiformes	Hexanchidae	*Hexanchus nakamurai*	2/0

*CITES-listed species with rtPCR species-specific assays.

**CITES-listed species without rtPCR species-specific assays.

We also tested our multiplex rtPCR protocol against processed shark fin samples collected in the Hong Kong retail markets (Table 2) in order to assess its applicability on highly degraded tissue samples. Only one of the nine species present in the multiplex was not tested on processed products (i.e., great white shark), since no samples for this species have been found after more than four years of extensive sampling in Hong Kong and mainland China (Cardeñosa *et al*. unpublished data). DNA extraction methods, rtPCR protocols, and thermal profiles followed the ones described above.

Three field tests were conducted in Hong Kong in collaboration with the Agriculture Fisheries and Conservation Department (AFCD). During these field tests the QuantStudio5 System, reagents, and plastic consumables were transported to local inspection facilities as a mobile lab. We used the following workflow during our field tests in Hong Kong. After AFCD personnel visually inspected the fins, tissue samples were taken and the QuantStudio5 System was used for Chelex extraction of DNA (~45 minutes), 5 μL of which was then added to the reaction plate. Two wells were used for a positive control using DNA from a previously known scalloped hammerhead sample and a negative control. Once the primer mix and SYBR Green were added, 35 PCR cycles were run with a posterior melt curve analysis (Supplementary Fig. S1). At the conclusion of the PCR and melt curve stages, the melt curve analysis was used to determine which species were present. The field tests involved (i) a container that had sacks of small, unprocessed fins (i.e., <10 cm across the base), (ii) an undeclared shipment of dried seafood that, among other products, included processed shark fins, and (iii) a container that was primarily carrying large unprocessed primary fins (i.e., first dorsal, pectorals, caudal). In the first two cases (i.e., small fins, processed fins) no positive visual identifications were made and we tested our rtPCR protocol against haphazardly selected fins. For the third case (i.e., large unprocessed primary fins), we tested our rtPCR protocol against fins identified as being from CITES listed species based on visual identification protocols (N = 21) and haphazardly selected fins that were thought not to be from these species (N = 35). Since Hong Kong authorities had not yet started to enforce the 2016 shark CITES listings at the time, the species-specific primers to detect the silky and thresher sharks were removed from the multiplex for these specific law-enforcement scenarios. For all of these cases a portion of the mitochondrial COI gene was later amplified and sequenced from unprocessed fins following the protocols by^31^, and from processed fins following the protocols by^15^ to assess concordance with the multiplex results. Sequences were compared to BOLD (FISH-BOL) and BLAST (GenBank) databases to identify them to the lowest taxonomic category possible.

## Results

**Figure 2:**
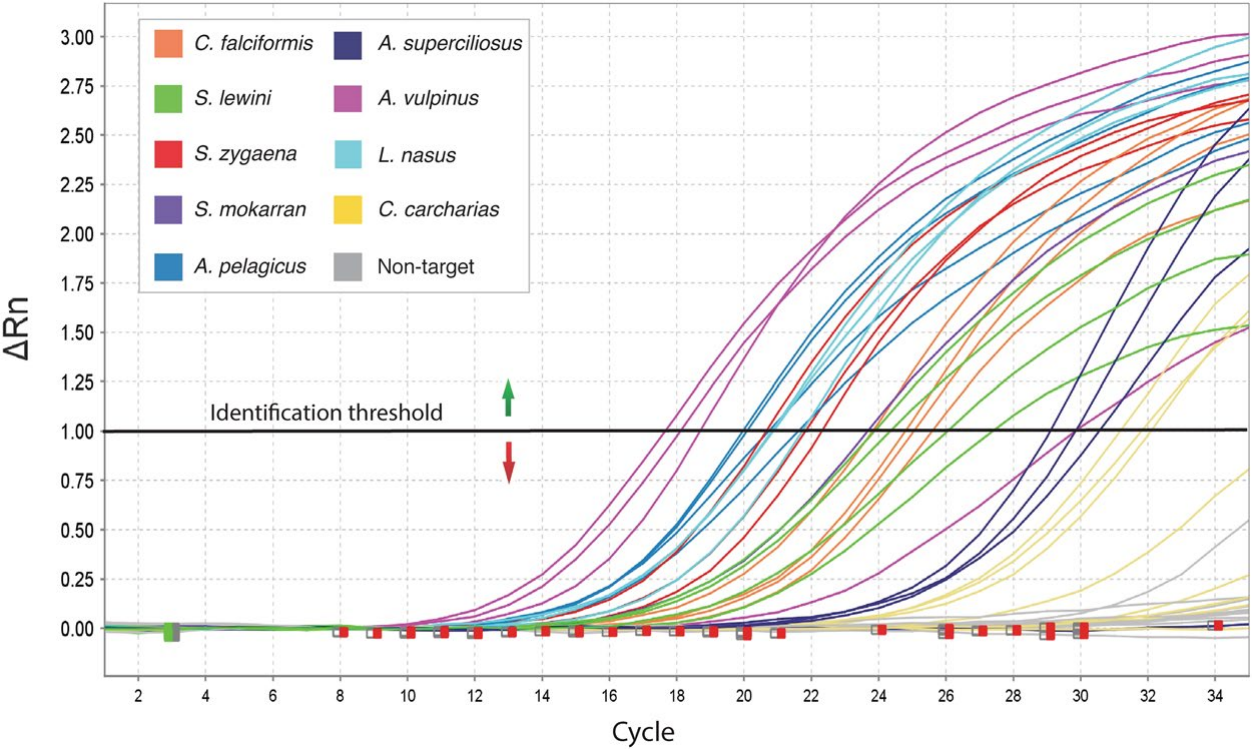
Amplification plot showing positive amplification of all CITES-listed species included in the multiplex rtPCR assay and negative amplifications of all non-target species tested. All samples below the identification threshold should not be considered positive.

**Figure 3:**
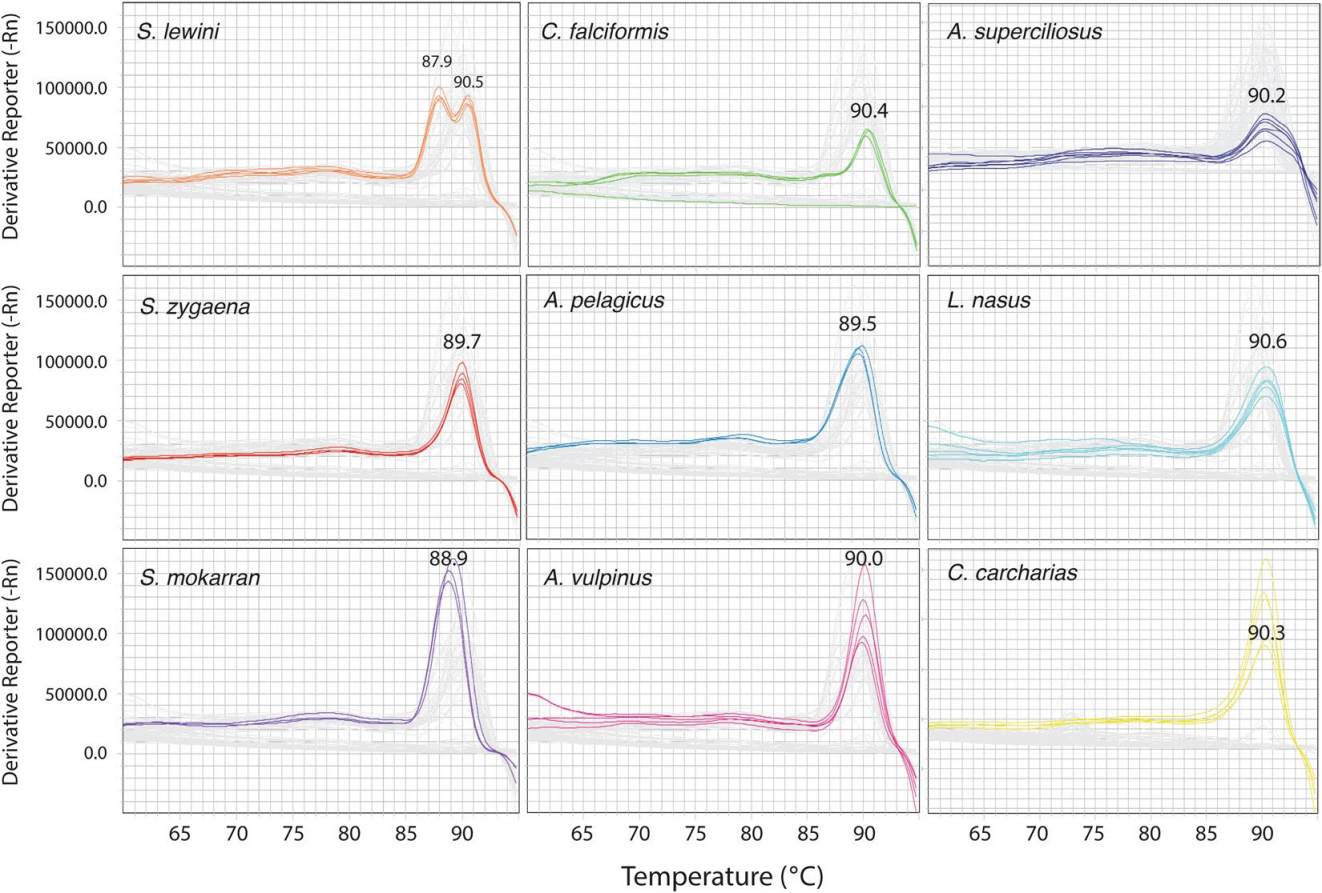
Melt curve plots for each CITES-listed species showing the differences between temperature and shapes between species.

Our putative silky shark specific primer performed well individually and in multiplex format, amplifying silky sharks from all over the world (Fig. 1). The final multiplex rtPCR assay containing this primer and the previously published primers for eight additional species correctly detected when any of these CITES-listed species were present and failed to amplify when using genomic DNA templates from all non-target species (Fig. 2). The multiplex proved to be highly reliable in this context, with a false positive rate of 0% with the test samples used (Table 2). There were no differences in the amplification profiles when primers were run individually or as a multiplex protocol. Melt curve temperatures for CITES-listed species occurred between 89° and 91 °C (Fig. 3), making the instantaneous post-PCR identification to species difficult. However, particular melt curve presented consistent shapes that were distinctive for certain species, enabling unambiguous post-PCR identification of *S*. *lewini* and *A*. *superciliosus* (Fig. 3). Our protocol was also able to consistently detect seven of the eight species tested on processed shark fin samples, with the exception of *A*. *superciliosus*, due to its large amplicon (i.e., around 1000 bps).

**Figure 4:**
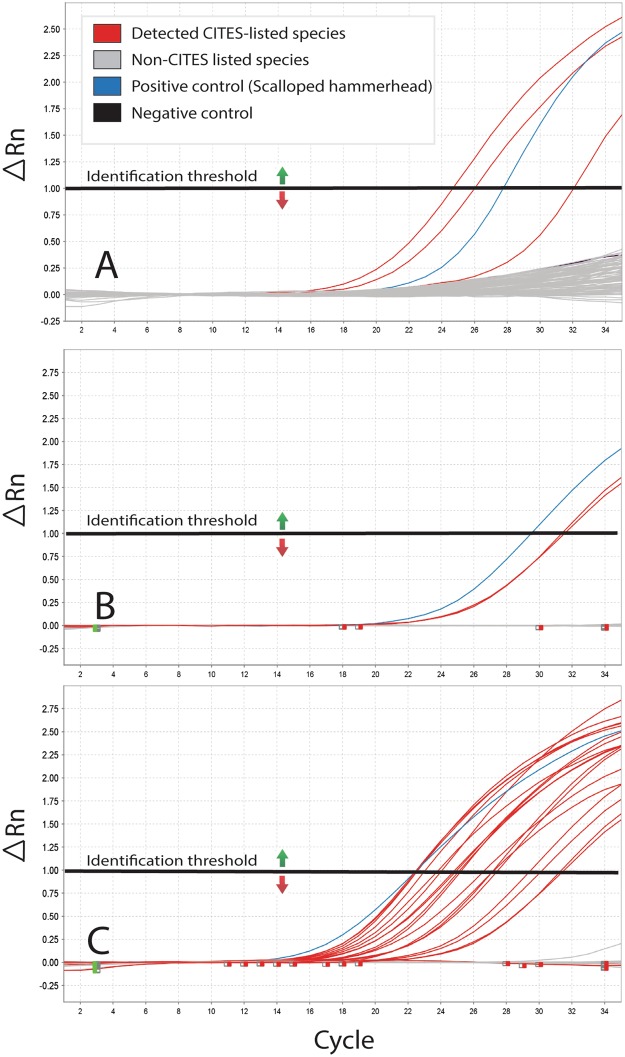
Amplification plots for the three field-based tests showing the detection of CITES listed species (red curves) among Non-CITES species (grey curves), a positive control (blue curve) and a negative control (black curve). (**A**) Shows the amplification plot of the small unprocessed fins case, (**B**) shows the amplification plot of the processed fins case, and (**C**) shows the amplification plot of the large unprocessed fins case.

The rtPCR protocol achieved positive identification of fins from CITES listed species (N = 24) across all three field tests in Hong Kong (Fig. 4). All positive results from the rtPCR were subsequently corroborated with DNA barcoding (i.e., there were no false positives). There were four false negative results out of a total of 118 fins tested from CITES listed species, involving one processed fin and three large unprocessed fins. Three of these not only failed with rtPCR but also with DNA barcoding and were only identified as CITES listed species using morphological guides. The barcoding analysis for the first case identified 77% of the samples as milk shark (*Rhizoprionodon acutus* [55%]), spinner shark (*C*. *brevipinna* [16%]), silky shark (*C*. *falciformis* [3%]) and scalloped hammerhead (*S*. *lewini* [3%]), the latter of which tested positive with the rtPCR. For the second case, species identification was achieved in 92.9% of the tested samples and were identified as blue shark (*P*. *glauca* [23.8%]), brownbanded bamboo shark (*Chiloscyllium punctatum* [21.4%]), bigeye thresher shark (*A*. *superciliosus* [19.1%]), scalloped hammerhead (*S*. *lewini* [7.1%, all but one of which had tested positive with the rtPCR]), white-spotted guitarfish (*Rhinchobatus australiae* [7.1%]), spottail shark (*C*. *sorrah* [4.8%]), whitecheek shark (*C*. *dussumieri* [4.8%]), blacktip shark (*C*. *limbatus* [2.4%]), and salmon shark (*L*. *ditropis* [2.4%]). Barcoding analysis identified 71.4% of the samples from the third case. All fins that tested positive with rtPCR were CITES-listed smooth hammerhead (*S*. *zygaena* [16.1%]), great hammerhead (*S*. *mokarran* [8.9%]), and scalloped hammerhead (*S*. *lewini* [7.1%]). All of them were correctly visually identified as being from CITES listed hammerheads by AFCD personnel. Three large fins marked as CITES-listed species during the visual inspection failed to amplify with both the rtPCR protocol and the barcoding analysis. Fins that had negative test results with rt-PCR later proved to be non-CITES listed tiger shark (*Galeocerdo cuvier* [7.1%]), blacktip shark (*C*. *limbatus* [3.6%]), snaggletooth shark (*Hemipristis elongata* [3.6%]), bull shark (*C*. *leucas* [1.8%]), silvertip shark (*C*. *albimarginatus* [1.8%]), and graceful shark (*C*. *amblyrhynchoides* [1.8%]). Barcoding also verified CITES listed oceanic whitetip (*C*. *longimanus* [7.1%]) and silky sharks (*C*. *falciformis* [1.8%]) that were not part of the multiplex.

## Discussion

Here, we present an easy-to-use, reliable, and to our knowledge, the fastest molecular protocol to date to detect the majority of CITES-listed shark species that are relatively common in the international trade (at least from the perspective of fins^9^). The multiplex used previously published and extensively tested PCR primers, adapting them to rtPCR format with SYBR Green chemistry. The only exception was the silky shark primer presented in^27^, which did not work well in this format. The novel silky primer we designed produced a smaller amplicon, promoting more efficient PCR and reliable detection. It amplified 96 known silky shark samples collected from all ocean basins where they occur and failed to amplify any other species, including 22 of 28 described congeners. We did not test the silky primer against all *Carcharhinus* species and none of the species that we lacked tissue samples for had a sequence in the National Center of Biotechnology Information (NCBI) GeneBank (http://www.ncbi.nlm.nih.gov/genbank/) that would enable us to check for primer mismatches that could prevent amplification. However, the untested *Carcharhinus* species (n = 6) are phylogenetically more distant to the silky shark than others that we have examined and presented negative results^33^ (Table 2). Therefore, it is unlikely that the novel primer amplifies other untested *Carcharhinus* species, especially at an annealing temperature of 69 °C. We also tested against all of the *Carcharhinus* species that are common in the fin trade, with the untested species occurring only rarely or not occurring at all^9^. Across all species our rtPCR multiplex generated 0% false positives during the testing phase and field trials. It is likely that the false positive rate of this multiplex will be zero or near zero because of the extensive testing of the published primers^16,17,27,28^ and the testing we conducted of the novel silky primer. Overall, we contend that the most probable source of false positives is contamination, which can be minimized by rinsing tissues with clean water prior to sampling and by using negative controls. A positive result from this protocol is robust evidence of the presence of one of these CITES listed species, which should be sufficient probable cause to at least hold the product for DNA barcoding that is currently widely accepted in an evidentiary context.

False negative results are unavoidable and no method, genetic or morphological, is entirely immune to them. With our rtPCR assay the frequency of false negative results could vary between individual runs according to the frequency of samples with low DNA quality (e.g., when tested on processed products) or the presence of PCR inhibitors. When the rtPCR multiplex was tested against processed shark fin samples, the bigeye thresher was the only CITES listed species included in the multiplex that failed to amplify, possibly due to the resulting large amplicon (i.e., >1000 bp)^15^. If false negatives are considered to be a major problem in law enforcement contexts we suggest that all test samples could be run twice: once with the rtPCR multiplex described and once with a protocol expected to amplify all shark products (e.g., a universal shark mini-barcode assay^34^ or universal ITS2 primers^27^). Samples that fail to amplify using both approaches could therefore be deemed inconclusive rather than registering as false negatives.

This protocol identifies nine out of twelve CITES-listed shark species, only omitting whale, basking, and oceanic whitetip sharks. The whale and basking sharks are relatively rare in the fin^9,10^ and meat trade and many of their fins are visually identifiable given their large size and other morphological characters^12^. The oceanic whitetip is more common in the contemporary shark fin trade^9,10^ but there are no published primers for this species. Examining the sequences for this species available on GenBank such as ITS2 and cytochrome oxidase I (COI) revealed very high sequence similarity (98%) between this and the dusky and Galapagos sharks (*C*. *obscurus*, *C*. *galapagensis*), which makes it extremely difficult to design a specific primer (see Shivji *et al*.^27^ who noted similar issues). While future efforts will explore other loci, the fins of oceanic whitetips have unique morphological characteristics (e.g. rounded apices with mottled white markings) that make them easily recognizable for law enforcement officers and inspectors^12^ and reduce the need for them to be in the present multiplex to monitor the fin trade. We conclude that this assay is currently ready-to-use for monitoring the fin trade given that the omitted species all have visually identifiable fins. It is ready-to-use for monitoring the trade of meat and other products, with the caveat that it does not amplify three CITES listed species, two of which are likely to be extremely rare^7,10^. Future work should aim to develop specific primers for these species that could be integrated into the present multiplex, although this may require surveys of additional loci.

The largest obstacle to routine screening of shark products is cost and time given large shipment volumes typical of the shark trade. The QuantStudio5 System and other rtPCR thermal cyclers have a relatively small footprint and are sufficiently portable, together with the laptop and all necessary reagents, pipettes, and disposable plastics, for this protocol to be deployed in ports or customs inspection areas as shown during our first field tests. The only on-site requisite is a source of electricity and cover from the elements. The speed at which this multiplex can detect these species is a key advance. We suggest the following workflow for sample identification using this multiplex, which can be completed in approximately 4 hours for 94 samples. After taking tissue samples (up to 94 for one run), the QuantStudio5 System can be used for Chelex extraction of DNA (~45 minutes), 5 μL of which is then added to all but one well of a 96 well reaction plate. Once the primer mix and SYBR Green are added, 35 PCR cycles should be run and positive amplifications (i.e., presence of CITES listed species) detected in wells that reach a conservative ΔRn value (i.e. the magnitude of the fluorescent signal generated by the PCR reaction) of 1.00 (Fig. 2), since no non-target species ever crossed that threshold during our testing with the optimized protocol. This threshold is normally reached in 60–70 minutes. We suggest reserving two wells, on a 96 well plate, for a positive control that includes DNA from a known CITES listed shark species in the multiplex that has previously amplified, and a negative control without a template. If the positive control sample fails to amplify it suggests the reagents are compromised. At the conclusion of PCR, the melt curve can be analyzed to determine which species is present (when possible; see Fig. 3), although the presence of any CITES listed species without the proper documentation is illegal and allows for the shipment to be seized and inspected in more detail for prosecution. Given this workflow, the concentrations of reagents and consumables used, the cost to screen one sample with the rtPCR multiplex was $0.94 USD.

Combining this multiplex with morphological identification guides^12^ enabled highly efficient and cost effective screening of imports at the border for the most common CITES listed sharks in trade from a wide range of potential fin products. The rtPCR protocol also proved to enhance the capacity of law-enforcement officers in Hong Kong to detect illicit trade. First, it achieved detection of CITES-listed species fins that otherwise went undetected because they were outside of the scope of morphological identification guides that are part of the AFCD inspection protocol (e.g., small fins and processed fins in the first and second cases respectively). In the third case it gave them near real time genetic confirmation of suspected CITES-listed shark fins that were flagged after their initial visual inspection, with at least 85.7% of their visual identifications that were tested proving to be accurate. The remainder failed to amplify with the rtPCR protocol and the barcoding analysis, probably due to degraded DNA or PCR inhibitors present in the extraction. Overall, our results highlight the need to implement this new rtPCR protocol to detect the CITES listed species in shipments of fins from small sharks, small secondary fin types (e.g., pelvic, anal, second dorsal fins), and processed fins that have been previously gone uninspected due to the lack of morphological identification protocols. All three cases contained CITES-listed shark fins that were exported without the required permits, aligning with recent evidence of CITES-listed species remaining among the most common in the contemporary shark fin trade in Hong Kong^10^. Also, the barcoding analysis of the COI locus allowed the confirmation of the CITES species detection by our protocol in all three cases.

The rtPCR approach described here can aid law enforcement officers in major shark trade hubs around the world meet their CITES requirements and can also serve as a model for other monitoring applications for sharks. For example, the International Commission for the Conservation of Atlantic Tunas (ICCAT) prohibits the retention, transhipment, landing, storing, or trade of the three hammerheads and bigeye thresher sharks^35^, which could be monitored and enforced by member states in port using a modified five-primer version of our multiplex assay. Domestic and regional prohibitions on other species await either the development of a specific multiplex or design of species-specific primers. Usually, rtPCR protocols for quantification purposes (e.g. gene expression^21^), or using TaqMan probes, are recommended for amplicon sizes of less that 200 bps. We demonstrated that the SYBR Green chemistry allows for amplification and detection of fragments as long as 1000 bps (Table 1), increasing the scope for finding an appropriate region to design many species-specific primers within a locus (e.g., ITS2) and tailor rtPCR assays for sharks according to the specific legal application. Given the widespread detection of illegal trade of meat and fins from sharks and their relatives^8,10,36–40^ it is imperative that we deploy all available tools, including portable rtPCR field assays, for better monitoring and enforcement of laws intended to protect them.

## Electronic supplementary material

Supplementary information
