# Supplementary material for: Multiplex real-time PCR assay to detect illegal trade of CITES-listed shark species
Source: Sci Rep. 2018 Nov 5;8:16313. doi: 10.1038/s41598-018-34663-6 (PMC6218538; doi:10.1038/s41598-018-34663-6)

Multiplex real-time PCR assay to detect illegal trade of CITES-listed shark species

Diego Cardeñosa[1,2*], Jessica Quinlan[3], Kwok Ho Shea[4], Demian D. Chapman[3]

[1] School of Marine and Atmospheric Science, Stony Brook University, Stony Brook, New York, 11794, United States of America

[2] Fundación Colombia Azul, Bogotá, Colombia

[3] Department of Biological Sciences, Florida International University, 3000 NE 151st Street, North Miami, Florida, 33181, United States of America

[4] BLOOM Association, Central, Hong Kong.

*Corresponding Author: Diego Cardeñosa

Email: diego.cardeosa@stonybrook.edu

Supplementary Figure S1. Thermal cycling profile for the multiplex rt-PCR indication the different stages, times, and temperatures. Camera icons represent the stages where the QuantStudio 5 system collects the data.

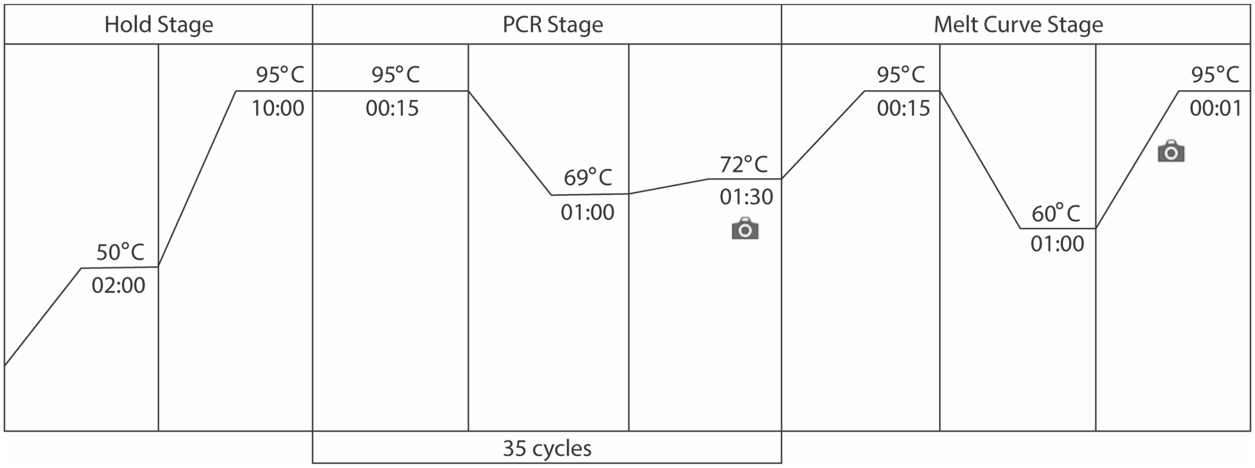

Supplement: Supplementary file 1 — Supplementary information [file 41598_2018_34663_MOESM1_ESM.pdf]
